# The effects of physical exercise with additional visual tasks on vision and anxiety in children aged 10–11 years

**DOI:** 10.3389/fpsyg.2025.1665603

**Published:** 2025-10-07

**Authors:** Guiming Zhu, Miyu Wang, Jingchi Wang, Pengfei Li, Limei Jiang, Haijie Shi, Rongbin Yin, Junjie Ding

**Affiliations:** ^1^School of Physical Education and Sports, Soochow University, Suzhou, China; ^2^School of Jewelry, West Yunnan University of Applied Sciences, Tengchong, Yunnan, China; ^3^Jiyang College, Zhejiang A&F University, Shaoxing, China

**Keywords:** physical exercise, visual tasks, visual acuity, anxiety, children aged 10–11

## Abstract

**Purpose:**

This study investigated the effects of physical exercise with additional visual tasks on anxiety and visual acuity in children aged 10–11 years, and analyzed the mediating role of visual acuity in this relationship.

**Methods:**

Fifth-grade students from an experimental elementary school in Suzhou were selected and randomly assigned into a control group (*n* = 81) and an experimental group (*n* = 80). The experimental group underwent 16 weeks of physical exercise with additional visual tasks, while the control group engaged in regular physical exercise. Uncorrected distance visual acuity (UDVA), kinetic visual acuity (KVA), and anxiety levels were measured both before and after the experiment.

**Results:**

Post-intervention, the experimental group showed significant improvements in left and right eyes UDVA and KVA (*P* < 0.01) and a significant reduction in total anxiety scores (*P* < 0.01). The control group exhibited significant improvements in left and right eyes UDVA (*P* < 0.05), but not in KVA (*P* > 0.05), with a significant reduction in anxiety scores (*P* < 0.01). Significant positive correlations were found between left and right eyes UDVA and KVA, and significant negative correlations between total anxiety scores and both left and right eyes UDVA and KVA. In the experimental group, KVA and left eye UDVA mediated the impact of physical exercise with additional visual tasks on anxiety.

**Conclusion:**

Physical exercise with additional visual tasks improved UDVA and KVA in children aged 10–11 and effectively reduced anxiety in fifth-grade students. KVA and left eye UDVA acted as chain mediators in this effect.

## 1 Introduction

Myopia has become one of the most serious health issues affecting humanity. Studies indicated that the prevalence of myopia in China surpasses that of other countries worldwide, with the rate among adolescents being particularly high (Deborah and Doerte, 2012). Myopia started to occur at a younger age and was rapid in progressing (Yan et al., 2017). A meta-analysis by Holden et al. (2016) predicted that by 2050, the prevalence of myopia will double from the year 2000 levels, potentially affecting nearly half of the global population. Of these, the number of people with high myopia will reach 938 million, which will account for 9.8 per cent of the global population. The etiology of myopia is complex and irreversible, significantly impacting the visual health of children and adolescents. Primary school is a critical period for growth and development, particularly for visual health. Research has indicated that China’s myopia rate has surpassed that of other countries worldwide, with adolescents exhibiting the highest rate of myopia (Deborah and Doerte, 2012). Furthermore, the onset age of myopia continues to decrease, and the progression of myopia is accelerating rapidly (Yan et al., 2017). In 2022, the overall prevalence of myopia among children and adolescents in China reached 51.9%. Among primary school students, the myopia rate was 36.7%, while it was 71.4% for junior high school students and 81.2% for senior high school students (The State Council of the People’s Republic of China, 2024). Research has indicated that upper primary to middle school has become a high prevalence stage for myopia (Ministry of Education of the People’s Republic of China, 2021). Children aged 10–11, who are in grade five of primary school, are undergoing a phase of rapid physical development while simultaneously facing the pressure of transitioning to junior high school, a period marked by a significant increase in homework load and prolonged periods of close-up visual tasks, leading to a more pronounced decline in vision (Gu et al., 2018). Furthermore, contemporary society is characterized by intensifying competition, with children and adolescents facing mounting pressures from social, educational and familial environments. Consequently, mental health risks among this demographic are escalating, making them increasingly susceptible to anxiety and depressive symptoms as they grow up, which may subsequently trigger a range of associated complications. Upper elementary students face academic pressures related to transitioning to junior high school, along with high parental expectations, social pressures, and competitive environments, often leaving them feeling exhausted and helpless. These stressors can result in a higher incidence of anxiety, characterized by a complex emotional state of worry in the face of imminent or potential threats (Huang, 2003). While normal levels of anxiety can serve as an adaptive signal triggering cognitive, physiological, and behavioral changes to help individuals cope with sudden situations or threats (Weinberger, 2001), excessive anxiety can be detrimental. A meta-analysis involving 80,879 participants found that the prevalence of anxiety symptoms among children and adolescents worldwide was 20.5% (Racine et al., 2021). The study by Deng et al. (2023) indicated that during the COVID-19 pandemic, the prevalence rates of depressive and anxiety symptoms among Chinese children and adolescents were both 31%. These findings have confirmed that myopia and anxiety have a serious impact on the physical and mental health and future development of children and adolescents, while emphasizing the need for preventive measures.

Numerous studies have demonstrated a correlation between myopia and anxiety, underscoring their significant and serious impact on the physical and mental health of children and adolescents. Currently, the proliferation of electronic devices and increased academic pressures have led to longer periods of close-up visual tasks among children and adolescents, potentially causing vision decline. In the early stages of vision deterioration, individuals may experience minor mental stress. Concurrently, students face heightened academic demands and social competitive pressures. When mental stress persists without relief, it may lead to autonomic nervous system dysfunction. The eyes are regulated by the autonomic nervous system, so excessive mental stress can easily cause ciliary muscle spasms, which will lead to reduced sensitivity and impaired accommodation function of the ciliary muscle, thereby further accelerating the progression of myopia. However, the rapid decline in vision may cause greater mental stress, thus creating a vicious cycle. As early as 1967, Rosanes (1967) discovered through Rorschach tests that compared to emmetropes, myopic individuals exhibited reduced motor activity and displayed non-specific anxiety and hostility, suggesting potential anxiety. Myopic students experience mental stress such as worry, anxiety, and even fear due to the negative impact of rapid vision deterioration on their daily lives and studies. Myopia is identified as one of the factors influencing trait anxiety levels in junior high school students (Lazarczyk et al., 2016). Yokoi et al. (2014) demonstrated that adolescents with myopia were more prone to anxiety compared to their non-myopic peers, with those having high myopia being more susceptible to depression. A review of the impact of myopia in the mental health of children and adolescents reported that in the child and adolescent population, myopia was more strongly associated with anxiety than with depression, which suggested that higher levels of anxiety and depression were associated with more severe refractive error (higher myopia) (You et al., 2022). Myopia not only impairs vision but also tends to induce psychological stress and anxiety in children and adolescents. Ignoring these symptoms of anxiety or depression can lead to more complex and severe mental disorders as children age (Dylan et al., 2018), and may increase the risk of developing harmful habits such as smoking and drinking (Lee et al., 2021). This continuous impact can hinder their academic performance, social interactions, and overall healthy development.

Physical exercise plays a crucial role in promoting both visual and psychological health. Engaging in outdoor physical activities can significantly reduce the prevalence of myopia among children and adolescents (Holton et al., 2021; Gareth et al., 2021). Yin et al. (2018) suggested that incorporating ciliary muscle regulation mechanisms into exercise regimens is an effective strategy for the prevention and treatment of myopia in children and adolescents. Experimental research by Yurova et al. (2017) confirmed that physical exercise can maintain existing vision levels and slow the progression of myopia. A longitudinal study by Fu (2022) on 551 third-grade students over 1 year indicated that comprehensive physical intervention can slow the progression of poor vision and myopia. Li et al. (2020), through a meta-analysis, demonstrated that a 16–24 week intervention period with a frequency of three sessions per week and each session lasting 60–90 min has a positive effect on improving vision, especially among elementary school students. Yan et al. (2022) suggested that active participation in outdoor activities or physical exercises positively can influence adolescents’ emotional regulation, social adaptation, and mental health, with longer durations of sustained exercise leading to greater improvements in psychological health. Children and adolescents are at the critical stage of visual and psychological development. Participation in physical exercise can enhance physical fitness, improve vision, and prevent myopia, while also alleviating anxiety, depression, and other negative emotions. Consequently, this contributes to the overall enhancement of children’s and adolescents’ physical and mental health.

Numerous studies on accommodation theory have suggested that the process of vision deterioration is accompanied by dysfunction of the ciliary muscle’s accommodative function (Zhu et al., 2012; Wu et al., 2015). Lu et al. (2020) suggested that the primary purpose of visual training is to exercise the eye muscles, and that ciliary muscle accommodation training is one of the main methods of visual training. Ciliary muscle accommodation training is a training method for myopia caused by overloaded near eye use and is effective for myopia and amblyopia. During the training process, the eye need to constantly switch between distance and near vision, and the ciliary muscle alternately contracts and relaxes to adjust the size of the refractive power, so that the image falls just in the center of the retina, thus improving the ciliary muscle’s adjusting ability. Ciliary spasm refers to a decrease in the ability of the ciliary muscle to reverse its adjustment. Ciliary spasms occur when the eyes are held close to objects for long periods of time. Combining physical exercise with ciliary muscle training for far-near vision regulation can effectively alleviate the strain on the eyes caused by prolonged fixation, thereby reducing eye fatigue. This integration enhances the training effects on the ciliary muscle (Cao et al., 2019), effectively stimulating and activating the ciliary muscle, alleviating ciliary muscle spasms, and improving the sensitivity and accuracy of ocular accommodation (You et al., 2022). Additionally, it also exercises the extraocular muscles and enhances the precision in pupil and retinal tracking of objects (Zhang et al., 2022), thereby improving kinetic visual acuity and uncorrected distance visual acuity, and enhancing the overall coordination and accuracy of the visual system. At the same time, studies have indicated that kinetic visual acuity exhibits a moderate positive correlation with uncorrected distance visual acuity (Sun et al., 2018). Physical exercises incorporating visual recognition tasks can enhance the accommodative capacity of the ciliary muscle, alleviate ciliary muscle tension, and promote improvements in kinetic visual acuity (Ciuffreda and Wang, 2004), thereby influencing uncorrected distance visual acuity. Engaging in physical exercise strengthens physical fitness and cardiopulmonary function, thereby reducing the risk of psychological disorders (Kandola et al., 2019). Research in cognitive neuroscience provided evidence that participating in physical activities may release endorphins (Dishman and O’Connor, 2009) and stimulate the growth of brain capillaries (Kleim et al., 2002), contributing to improved psychological health.

Currently, studies both domestically and internationally have found that physical exercise promotes visual and psychological health in children. However, the mediating role of children’s vision in the effect of physical exercise on alleviating anxiety has not been thoroughly investigated. Therefore, based on the aforementioned theoretical foundation, this study hypothesizes that children’s vision mediates the impact of physical exercise on anxiety. This hypothesis will be tested through experimental research to validate the mediating effect of children’s vision on the relationship between physical exercise and anxiety.

## 2 Experimental subjects and methods

### 2.1 Experimental subjects

This study employed a cluster randomization method based on practical considerations such as school administration. Using all fifth-grade students at a primary school in Suzhou as the sampling frame, four fifth-grade classes were randomly selected. Subsequently, a researcher from the study team who would not participate in subsequent interventions or evaluations assigned these four classes numbers 1–4 and randomly divided them equally into two groups. The generated random allocation sequence was sealed in opaque envelopes and remained unopened until baseline data collection was completed. Another researcher then opened the envelopes and implemented the allocation to ensure the fairness of the process.

Based on this study a 2 × 2 repeated measures ANOVA was used. The sample size was calculated using a G*Power analysis (Computer statistical soft-ware version 3.1.9.7^1^), with α = 0.05, a power value of 0.9, and a medium effect size. It was ultimately concluded that this sample size required a minimum of 44 subjects to determine the statistical significance of the intervention. Thus, 161 children aged 10–11 years were selected as subjects for this study. The students were randomly assigned to either a control group (*n* = 80; Class 5-1 and Class 5-2) or an experimental group (*n* = 81; Class 5-3 and Class 5-4). The intervention period lasted 16 weeks, with sessions occurring three times per week, each lasting 40 min. The control group engaged in regular physical exercise, while the experimental group performed physical exercise with additional visual tasks. Specific information about the participants can be found in Table 1.

**TABLE 1:** Distribution of experimental subjects (N = 161).

Group	Male	Female	Total
Experimental group	41	40	81
Control group	41	39	80
Total	82	79	161

**FIGURE 1:**
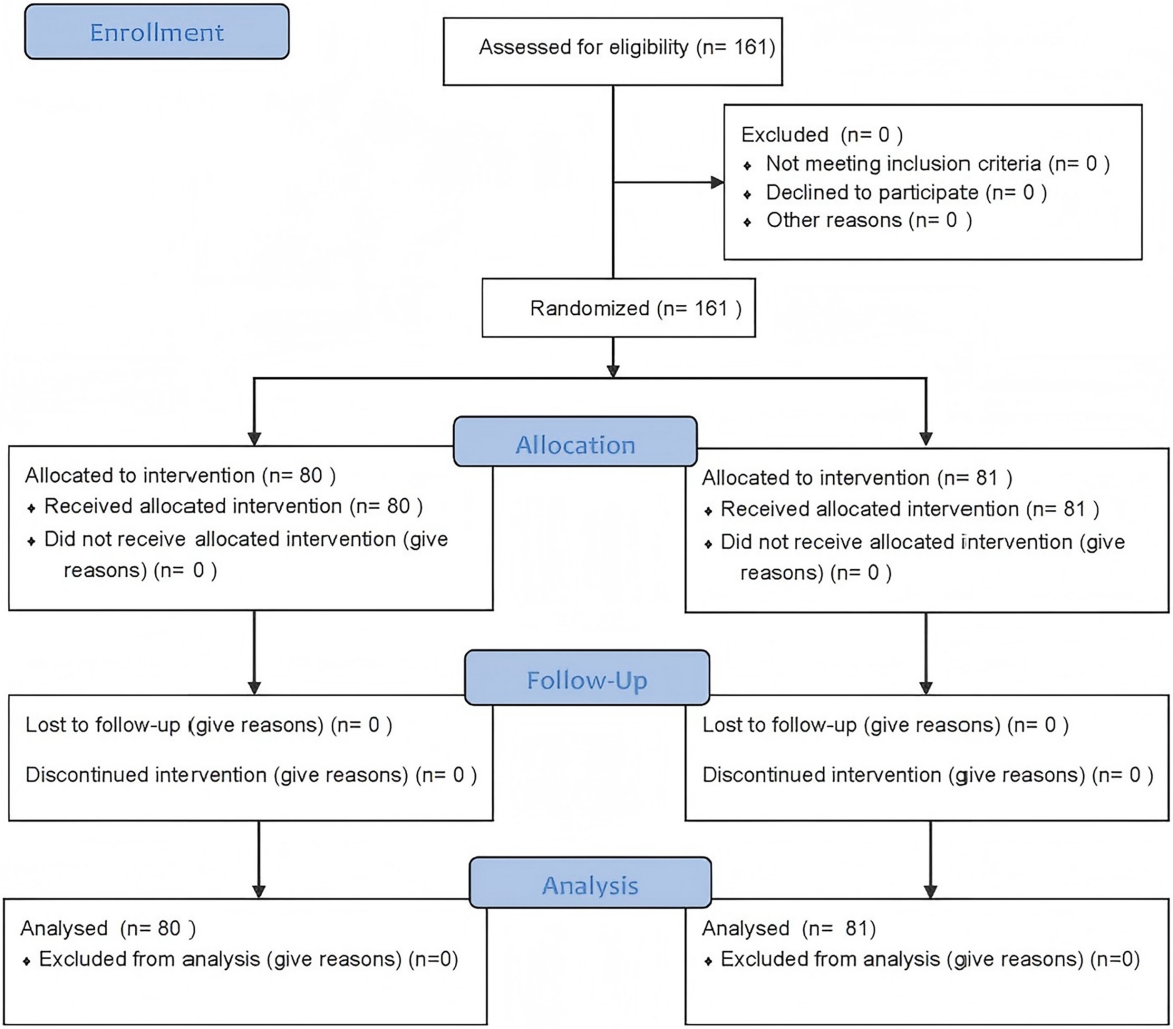
CONSORT flowchart.

Participant selection criteria were as follows: (1) Fifth-grade students who are physically and mentally healthy with no impairments affecting physical exercise. (2) Students without any severe structural diseases of the organs, and without astigmatism, amblyopia, or keratitis. (3) Students not wearing orthokeratology lenses (OK lenses). (4) Uncorrected distance visual acuity (UDVA) ≥ 4.0. The study protocol was in accordance with the Helsinki Declaration and was approved by the ethics committee of the first author’s university (No. SU-DA20201010H01). In this study, the informed consent form was distributed to the parents before the commencement of the experiment and the experimental procedure was agreed upon by the guardians of the subjects. The CONSORT flowchart for this study was shown in Figure 1.

### 2.2 Experimental plan

This study employed physical exercises with additional visual tasks, incorporating the principles of ciliary muscle accommodation training. Dynamic visual tasks, which require alternating near and far vision, were integrated into the exercises. The research indicates that incorporating visual training into physical exercises, with dynamic visual stimuli presented for 3 s, yields the best results for improving elementary students’ vision (Zhou et al., 2023). Visual acuity can be categorized into uncorrected distance visual acuity (UDVA) and kinetic vision acuity (KVA). Uncorrected distance visual acuity (UDVA) refers to the measured vision that has not been corrected by any optical lens. Kinetic vision acuity (KVA) refers to the eye’s ability to perceive details of moving objects. Different frequencies of ciliary muscle exercises have varying effects on students’ vision: high-frequency exercises (60 times) significantly enhance UDVA, mid-frequency exercises (30 times) better improve KVA, and low-frequency exercises (15 times) slow the rate of vision deterioration (Yin et al., 2022b). Therefore, this study designed an experimental protocol that included 30 repetitions of near-far vision alternation with a 3 s presentation time, scientifically integrating visual tasks into ball games and physical education activities.

At the age of 10–11, children are in a critical period of mental health development and a sensitive stage of physical growth. The experimental content was designed according to the physical growth patterns and psychological development characteristics of fifth-grade students. This was done in strict accordance with the requirements of the “Compulsory Education Stage Physical Education and Health Curriculum Standards (2022 Edition)” and the school’s teaching requirements. The teaching content primarily included ball sports such as basketball and soccer, as well as physical fitness games. The basketball activities included stationary high dribbling, dribbling while moving, chest passes and catches, and passing and catching while moving. The soccer activities included dribbling with the top of the foot, inside foot passes and catches, and passing and catching while moving. The physical fitness games included three-legged relay races, jumping relays, and tag with number calling. The specific details are provided in Table 2. Additionally, to ensure students can follow the training plan, the teacher provided detailed explanations and demonstrations of each exercise before practice sessions to facilitate understanding. Throughout each experiment, the teacher circulated to offer guidance and remind students to practice diligently, utilizing tools such as whistles to assist the exercises.

**TABLE 2:** Design of the intervention program content.

Project	Exercise content	Exercise frequency	Exercise time	Ciliary muscle adjustment training design
Basketball	Stationary high dribbling, dribbling while moving, chest passes and catches, passing and catching while moving	Three times a week, with each session lasting 40 min.	For a total of 16 weeks	Based on the principles of ciliary muscle regulation training, dynamic visual tasks that alternate between near and far vision were incorporated into physical exercise. The control group did not receive ciliary muscle intervention and participated in regular physical exercise. The experimental group integrated ciliary muscle training into each class’s exercises, with each task lasting 3 s and a frequency ranging from 30 to 60 times.
Soccer	Dribbling with the top of the foot, inside foot passes and catches, dribbling with the inside foot + shooting			
Games	Three-legged relay races, jumping relays, tag with number calling, strongest transporter			

During the experiment, students in the experimental group (engaging in physical exercise with additional visual tasks) were required to complete binocular identification tasks involving varied distances during their exercise sessions. Students in the experimental group performed the recognition tasks primarily by looking at specialized vision cards. The control group students participated in regular physical exercise. This experiment began on 04-09-2023 and ended on 05-01-2024. The intervention period lasted for 16 weeks, with interventions occurring three times per week, each session lasting 40 min. In order to try to exclude the interference of other factors on the experimental results, the following measures were used to control the experimental variables in this experiment to ensure the accuracy and reliability of the research results. Students from four different classes were randomly assigned to experimental and control groups. Two administrative classes were the experimental group and two administrative classes were the control group. In the course of the experiment, the experimental group and the control group maintained uniformity in terms of the exercise field, exercise time, and exercise program in each group, except for the additional visual conditioning training task, in order to ensure the rigor of the experiment. From the beginning to the end of the experiment, all teaching duties were carried out by the same person to ensure consistency of the variables and to minimize the influence of external factors on the experiment. At the same time, strict attendance records were maintained throughout the experiment, and students who took excessive leave or completed too many internships were ultimately excluded to ensure the validity of the experiment.

### 2.3 Testing methods

Data collection for uncorrected distance visual acuity (UDVA), kinetic visual acuity (KVA), and the Screen for Child Anxiety Related Emotional Disorders (SCARED) was conducted before the intervention (Week 1) and after the intervention (Week 17). In this experiment, two tests were conducted, pre-test (T1: September 2023) and post-test (T2: January 2024) and questionnaires were administered twice to the same subjects and returned. The testing took place in the gymnasium of an experimental elementary school in Suzhou, where the environment was quiet and well-lit. To minimize interference from other factors and ensure the accuracy and reliability of the research results, the following measures were implemented to control experimental variables. UDVA and KVA tests, along with data collection, were conducted by a specialized professional team, with testing environments adhering to standard protocols. The test site had well-lit conditions and no other noise interference. Before beginning the test, students would line up in order of their student ID numbers. During the test, students would take the exam in sequence, while test administrators maintained order in the testing areas and reminded students to remain quiet. The completion of the SCARED was strictly supervised, with a 16-week interval between the two administrations to ensure the accuracy of the responses.

#### 2.3.1 SCARED test

This study utilized the Screen for Child Anxiety Related Emotional Disorders (SCARED). The Screen for Child Anxiety Related Emotional Disorders (SCARED) is a widely used psychometric instrument developed by Birmaher in 1997. This self-report scale is designed for assessing anxiety disorders in children and adolescents aged 8–18. Factor analysis of the scale identified five dimensions: somatic/panic, generalized anxiety, separation anxiety, social phobia, and school phobia. The scale was revised to 41 items in 1999 and further adapted in 2008 by Su Linyan, Wang Kai, and others from the Second Xiangya Hospital of Central South University, establishing national norms for China. The SCARED has demonstrated good test-retest reliability and validity for assessing anxiety symptoms in Chinese children (Wang et al., 2002). It employs a three-point Likert scale: 0 = not true, 1 = sometimes true, and 2 = often true. A total score of 23 or higher indicates the potential presence of an anxiety disorder, with higher scores reflecting greater severity of anxiety. The SCARED questionnaires were distributed and completed on a class-by-class basis. Prior to commencing the assessment, professional test administrators provided explanations to students to facilitate their understanding of the items. Concurrently, test administrators supervised the completion process and addressed any queries raised by students during the questionnaire completion.

Before the intervention, the reliability and validity of SCARED were assessed. The results are presented in Tables 3, 4. The Cronbach’s alpha coefficients for the overall anxiety scale and its dimensions were all greater than 0.7, indicating good internal consistency among the items and, consequently, high reliability of the scale. The Kaiser-Meyer-Olkin (KMO) value was 0.925 (greater than 0.7), and the Bartlett’s test of sphericity was significant (*p* < 0.05), further confirming the validity of the scale. These findings are consistent with the reliability and validity results of the SCARED urban norms in China (Wang et al., 2002).

**TABLE 3:** Reliability test results of the anxiety disorder screening scale before experiment.

Dimensional division of the scale	Items	Cronbach’s alpha
Somatic/panic symptoms	13	0.942
Generalized anxiety	9	0.955
Separation anxiety	8	0.910
Social phobia	7	0.854
School phobia	4	0.830
Overall anxiety scale	41	0.961

**TABLE 4:** Validity test results of anxiety emotional disorder screening form before experiment.

Overall anxiety emotional disorder screening form		
KMO (Kaiser-Meyer-Olkin) sampling adequacy measure		0.925
Bartlett’s sphericity test	Approximate Chi-square	4964.069
	Degrees of freedom	820
	Significance	0.000

#### 2.3.2 UDVA test

The uncorrected distance visual acuity (UDVA) was measured using the Standard for Logarithmic Visual Acuity Charts (GB11533), with all procedures strictly adhering to the relevant standards. Before each test, the visual acuity chart light box was placed on a level tabletop and its brightness was verified to be functioning properly. A five-point recording method was used to document the UDVA of both the left and right eyes, with the measurement range between 4.0 and 5.3. During testing, each line is tested sequentially from top to bottom. Except for the first line, at least two letters must be tested per line until the student can no longer see clearly. The Standard for Logarithmic Visual Acuity Charts (GB11533) is the common and authoritative UDVA measurement method in China, which has a clear definition of the visual acuity range.

#### 2.3.3 KVA test

Kinetic visual acuity (KVA) was measured using the XP.14-TD-J905 KVA tester manufactured by Shanghai Tuofeng Automation Technology Co., Ltd., in accordance with the Chinese National Standard GB 18463-2001. This device assesses the dynamic visual ability of the test subject by simulating a moving target. At the same time, a computer was used to automatically collect data and display the results during the test. Participants were seated in front of the device, with their forehead positioned against the top edge of the viewing hole and their nose against the lower edge, eyes open, and hands on the joystick below the viewing hole. The test commenced upon activation by the tester’s card swipe. Participants observed a letter “C” moving rapidly from 50 m away at a speed of 30 km/h, increasing in size. They were required to identify the direction of the gap in the “C” and move the joystick in that direction (up, down, left, or right) to complete the round. Testers recorded the specific values for each round. Three rounds were conducted with 30 s intervals between each, and the average value of the three rounds was used as the final KVA score. The test values ranged from 0.1 to 1.6, with higher values indicating better KVA. Additionally, the difference between any two of the student’s three test scores must not exceed 0.3 points. If any of the above situations occur, retesting should be conducted.

### 2.4 Date analysis methods

After entering the pre- and post-experiment data, SPSS 26.0 was first used to test the reliability and validity of the Screen for Child Anxiety Related Emotional Disorders (SCARED). The Harman single-factor test was then employed to check for common method bias. Subsequently, SPSS was utilized for homogeneity testing and descriptive statistics of kinetic visual acuity (KVA), uncorrected distance visual acuity (UDVA) for both eyes, and SCARED scores. Repeated measures ANOVA was used to analyze changes in SCARED scores, the dimensions of the SCARED, KVA, and UDVA for both eyes. Finally, Pearson correlation analysis and mediation effect testing using Hayes’ PROCESS Model 6 in SPSS were conducted. Parameter estimation employed the bootstrap method, with 5,000 resamples based on the original sample, and significance was determined if the 95% confidence interval did not include zero.

Furthermore, strict attendance records were maintained throughout the experiments and tests, documenting each student’s attendance rate and number of test attempts. Concurrently, students with excessively low attendance rates or who did not participate in all tests were excluded. As this study was conducted within the school’s physical education curriculum, maintaining the students’ established school attendance and class schedules, few instances of the aforementioned circumstances arose, thereby ensuring an adequate sample size.

## 3 Results

### 3.1 Changes in UDVA, KVA, and SCARED scores between the experimental and control groups

To investigate the impact of physical exercise with additional visual tasks on the vision and anxiety of children aged 10–11, a 2 × 2 repeated measures ANOVA was conducted on the UDVA, KVA, and SCARED total scores for both the experimental and control groups. The results are presented in Tables 5, 6.

**TABLE 5:** Intergroup comparison of relevant test indicators between the two groups of students at T1 and T2 stages.

Indicators	Time	Group (I)	Group (J)	Mean difference (I-J)	t-value	*P*-value
UDVA (right eye)	T1	Control group	Experimental group	−0.025	−0.608	0.544
	T2	Control group	Experimental group	−0.254	−6.176	0.000
UDVA (left eye)	T1	Control group	Experimental group	−0.008	−0.211	0.833
	T2	Control group	Experimental group	−0.242	−6.808	0.000
KVA	T1	Control group	Experimental group	0.011	0.375	0.708
	T2	Control group	Experimental group	−0.278	−9.356	0.000
Total score of anxiety scale	T1	Control group	Experimental group	0.673	0.238	0.812
	T2	Control group	Experimental group	12.569	4.440	0.000

**TABLE 6:** Intragroup comparison of relevant test indicators within the two groups of students at T1 and T2 stages.

Group	Indicators	T1 (I)	T2 (J)	Mean difference (J)	t-value	*P*-value
Control group	UDVA (right eye)	4.777 ± 0.243	4.845 ± 0.322	−0.067	−2.872	0.005
	UDVA (left eye)	4.791 ± 0.282	4.873 ± 0.207	−0.081	−2.902	0.004
	KVA	0.367 ± 0.218	0.418 ± 0.129	−0.050	−2.137	0.034
	Total score of anxiety scale	37.500 ± 19.589	33.112 ± 19.224	4.387	3.338	0.001
Experimental group	UDVA (right eye)	4.802 ± 0.291	5.099 ± 0.158	−0.296	−12.687	0.000
	UDVA (left eye)	4.799 ± 0.249	5.115 ± 0.140	−0.316	−11.359	0.000
	KVA	0.356 ± 0.209	0.696 ± 0.184	−0.339	−14.455	0.000
	Total score of anxiety scale	36.827 ± 18.326	20.543 ± 14.222	16.284	12.467	0.000

**FIGURE 2:**
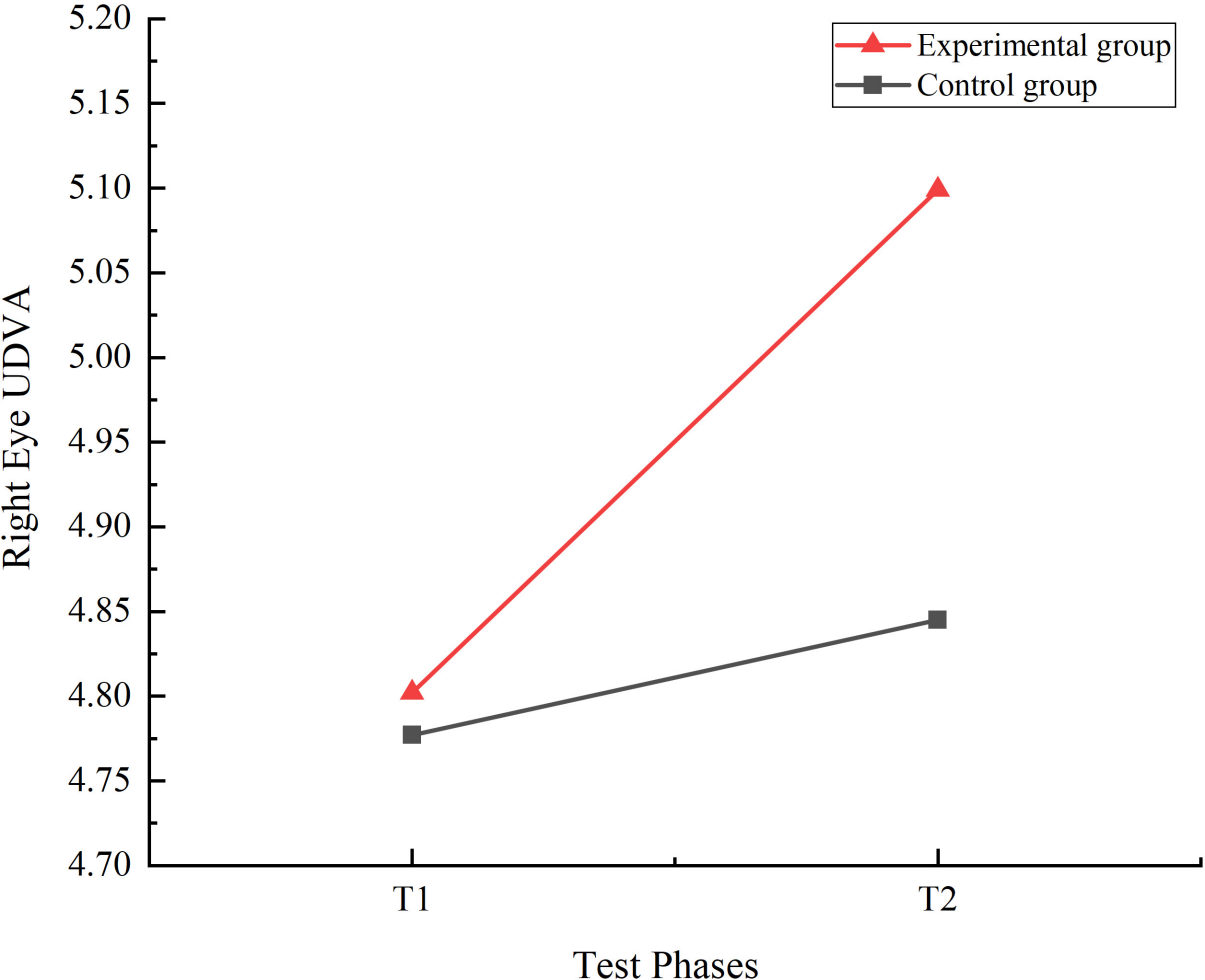
Trend chart of right eye uncorrected distance visual acuity changes for two groups of students in phases T1 and T2.

**FIGURE 3:**
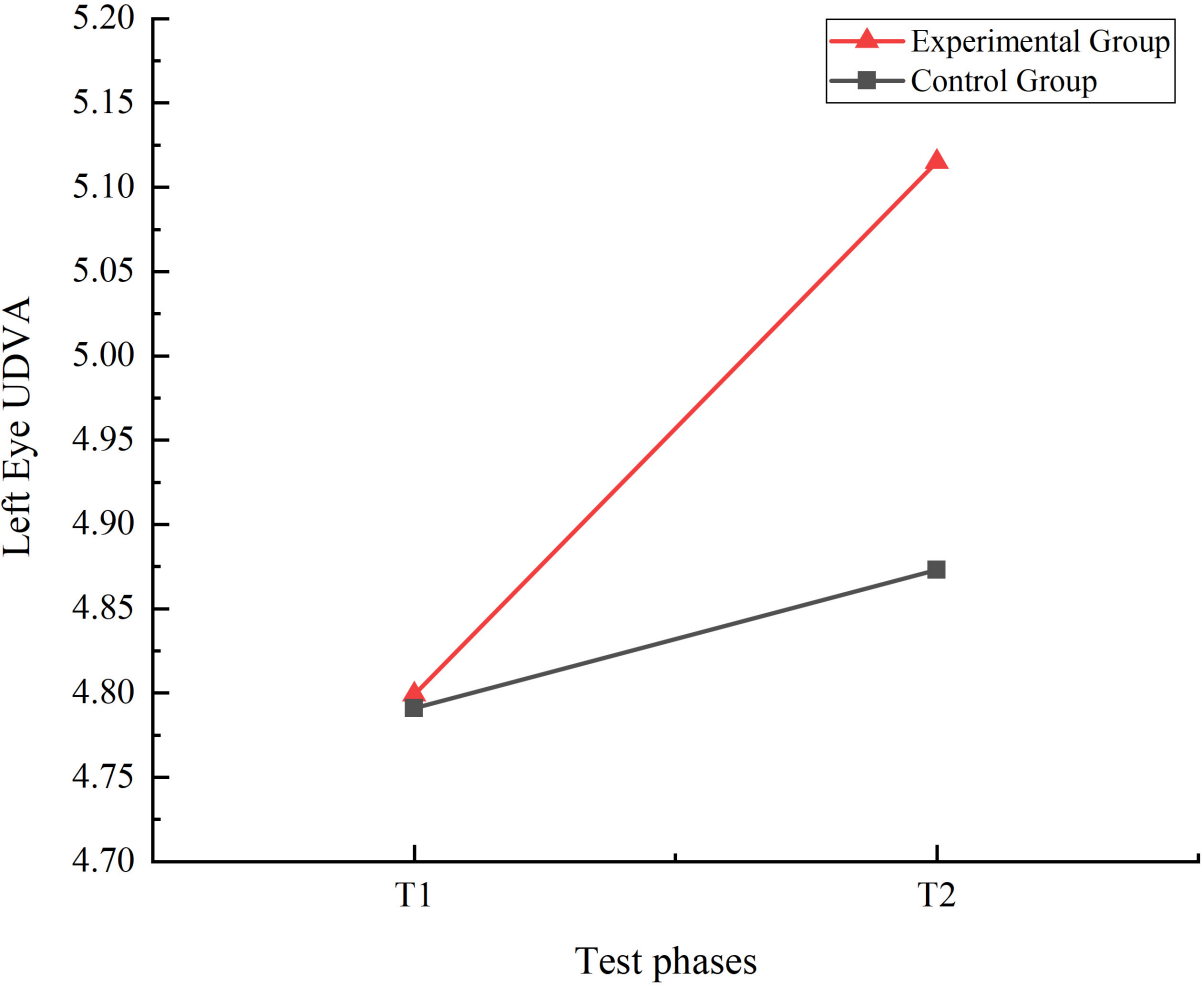
Trend chart of left eye uncorrected distance visual acuity changes for two groups of students in phases T1 and T2.

A 2 (Time: Pre-test, Post-test) × 2 (Group: Experimental, Control) repeated measures ANOVA was conducted to analyze the UDVA of the left and right eyes at T1 and T2 stages for both the experimental and control groups. The results of the analyses were expressed using F-values. The F-value is the ratio of the mean square treatment to the mean square error. The results of this experimental analysis were expressed as F (degrees of freedom between groups, degrees of freedom within groups) = specific values. The results indicated significant main effects for the group factor [right eye: F(1, 159) = 13.739, η_p_^2^ = 0.080, *P* = 0.000, *P* < 0.01] [left eye: F(1, 159) = 17.784, η_p_^2^ = 0.101, *P* = 0.000, *P* < 0.01] and the time factor [right eye: F(1, 159) = 120.574, η_p_^2^ = 0.431, *P* = 0.000, *P* < 0.01] [left eye: F(1, 159) = 101.308, η_p_^2^ = 0.389, *P* = 0.000, *P* < 0.01]. Additionally, the interaction effect between time and group was significant [right eye: F(1, 159) = 47.691, η_p_^2^ = 0.231, *P* = 0.000, *P* < 0.01] [left eye: F(1, 159) = 35.384, η_p_^2^ = 0.182, *P* = 0.000, *P* < 0.01]. Simple effect analysis revealed no significant differences in UDVA for both the right and left eyes between the control and experimental groups at T1 right eye: I-J = −0.025, *P* = 0.544, *P* > 0.05) (left eye: I-J = −0.008, *P* = 0.833, *P* > 0.05). However, at T2, there were highly significant differences in UDVA for both eyes between the two groups (right eye: I-J = −0.254, *P* = 0.000, *P* < 0.01) (left eye: I-J = −0.242, *P* = 0.000, *P* < 0.01). Both the control group (right eye: I-J = −0.067, *P* = 0.005, *P* < 0.05) (left eye: I-J = −0.081, *P* = 0.004, *P* < 0.05) and the experimental group (right eye: I-J = −0.296, *P* = 0.000, *P* < 0.01) (left eye: I-J = −0.316, *P* = 0.000, *P* < 0.01) showed significant improvements in UDVA for both eyes from T1 to T2. The trends in UDVA for the two groups were shown in Figures 2, 3.

**FIGURE 4:**
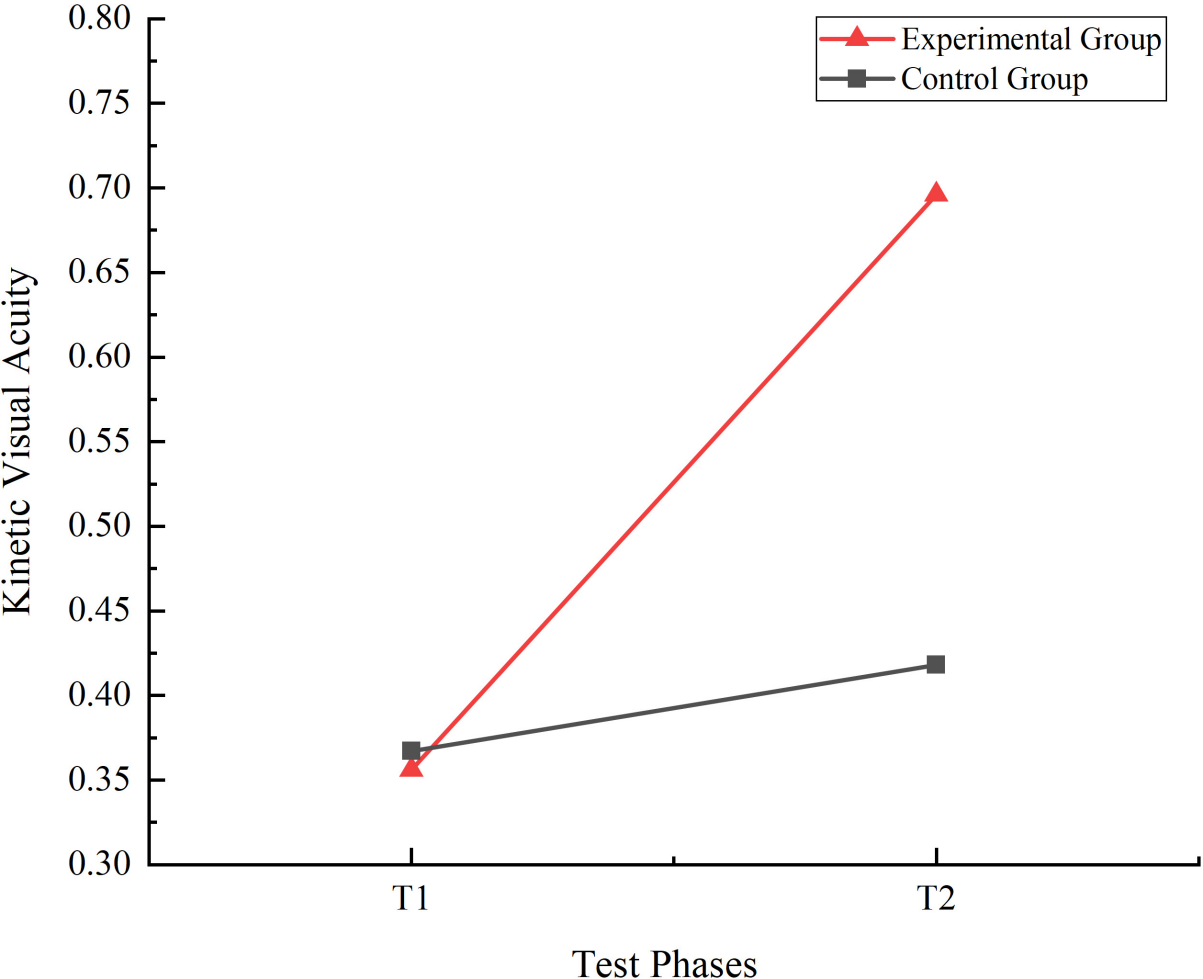
Trend chart of kinetic visual acuity changes for two groups of students in phases T1 and T2.

A 2 (Time: Pre-test, Post-test) × 2 (Group: Experimental, Control) repeated measures ANOVA was conducted to analyze the KVA of students in both the experimental and control groups at T1 and T2 stages. The results revealed significant main effects for the group factor [F(1, 159) = 29.420, η_p_^2^ = 0.156, *P* = 0.000, *P* < 0.01] and the time factor [F(1, 159) = 137.003, η_p_^2^ = 0.463, *P* = 0.000, *P* < 0.01]. Additionally, the interaction effect between time and group was significant [F(1, 159) = 75.231, η_p_^2^ = 0.321, *P* = 0.000, *P* < 0.01]. Simple effect analysis indicated that no significant differences in KVA between the control and experimental groups at T1 (I-J = 0.011, *P* = 0.708, *P* > 0.05). However, at T2, significant differences in KVA were observed between the two groups (I-J = −0.278, *P* = 0.000, *P* < 0.01). Furthermore, the KVA of control group students significantly improved from T1 to T2 (I-J = −0.050, *P* = 0.034, *P* < 0.05), while the experimental group students showed a highly significant improvement (I-J = −0.339, *P* = 0.000, *P* < 0.01). The trends in KVA for the two groups were shown in Figure 4.

**FIGURE 5:**
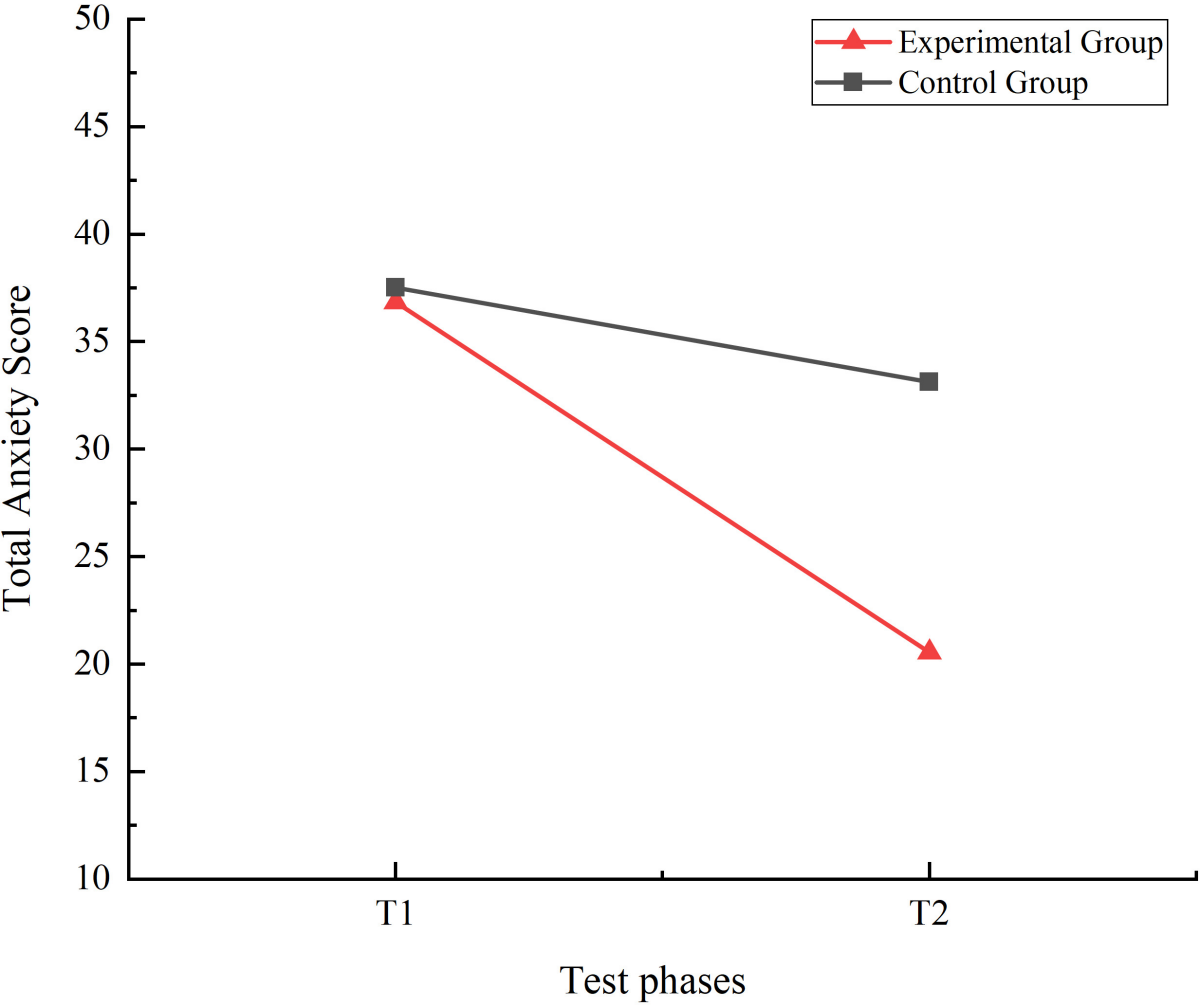
Trend chart of total anxiety score changes for two groups of students in phases T1 and T2.

A 2 (Time: Pre-test, Post-test) × 2 (Group: Experimental, Control) repeated measures ANOVA was performed to evaluate the total scores on the Children’s Anxiety Disorder Scale for both the experimental and control groups at T1 and T2. The analysis revealed significant main effects for both the group factor [F(1, 159) = 6.127, η_p_^2^ = 0.037, *P* = 0.014, *P* < 0.05] and the time factor [F(1, 159) = 124.449, η_p_^2^ = 0.493, *P* = 0.000, *P* < 0.01]. Additionally, the interaction effect between time and group was significant [F(1, 159) = 41.218, η_p_^2^ = 0.206, *P* = 0.000, *P* < 0.01]. Simple effect analysis indicated no significant differences in anxiety scale scores between the control and experimental groups at T1 (I-J = 0.673, *P* = 0.812, *P* > 0.05). However, at T2, significant differences were observed between the two groups (I-J = 12.569, *P* = 0.000, *P* < 0.01). Both the control (I-J = 4.387, *P* = 0.001, *P* < 0.01) and experimental groups (I-J = 16.284, *P* = 0.000, *P* < 0.01) showed significant differences in their anxiety scale scores from T1 to T2. The trends in total anxiety score for the two groups were shown in Figure 5.

### 3.2 Correlation analysis of UDVA, KVA, and anxiety in 10–11-year-old children

The Pearson correlation analysis of the various test indicators is shown in Table 7. The correlation analysis for this experiment employed the 95% confidence interval, with results expressed as r values. The r-value refers to the correlation coefficient between the variables in the sample and indicates the magnitude of the correlation. There was a significant positive correlation between right eye UDVA and left eye UDVA (r = 0.576, *P* < 0.01). KVA was significantly positively correlated with left eye UDVA (r = 0.369, *P* < 0.01) and right eye UDVA (r = 0.328, *P* < 0.01). The total score of the anxiety scale was significantly negatively correlated with right eye UDVA (r = −0.248, *P* < 0.01), left eye UDVA (r = −0.224, *P* < 0.01), and KVA (r = −0.327, *P* < 0.01).

**TABLE 7:** Correlation analysis of left and right uncorrected distance visual acuity (UDVA), kinetic visual acuity (KVA), and anxiety.

Indicators	UDVA (right eye)	UDVA (left eye)	KVA	Total score of anxiety scale
UDVA (right eye)	1	–	–	–
UDVA (left eye)	0.576**	1	–	–
KVA	0.328**	0.369**	1	–
Total score of anxiety scale	−0.284**	−0.224**	−0.327**	1

**Indicates *P* < 0.01, representing a highly significant difference.

### 3.3 Mediating effects of physical exercise with visual tasks on vision and anxiety

To investigate the mediating effects of physical exercise with additional visual tasks on vision and anxiety, a Pearson correlation analysis was conducted for the anxiety scale scores, uncorrected distance visual acuity (UDVA) of the left and right eyes, and kinetic visual acuity (KVA) at T1 and T2 stages. The analysis revealed significant correlations between anxiety scores and UDVA, as well as KVA, fulfilling the statistical requirements for mediation analysis. First, a dummy variable was created to represent the experimental groups, coding the group with additional visual tasks as 1 and the regular physical exercise group as 0. The mediation effect was then tested using Hayes’ PROCESS macro for SPSS, specifically Model 6. Parameter estimates were obtained using the bootstrap method with 5,000 resamples and a 95% confidence interval. In this analysis, the dummy variable was used as the independent variable (predictor), the total anxiety score as the dependent variable (outcome), and gender as the control variable. KVA and UDVA served as the mediating variables. The change in scores from T1 to T2 (i.e., T2 score minus T1 score) was used for the anxiety scale, KVA, and UDVA in the calculations.

#### 3.3.1 Regression analysis of physical exercise with additional visual tasks, KVA, left eye UDVA, and total anxiety score

A linear regression analysis was conducted with physical exercise with additional visual tasks as the independent variable and the change in KVA (difference scores) as the dependent variable. The results of the analysis were expressed here in terms of F-values, t-values and Beta-values. The F-value refers to the test of variance, which is an overall test of the entire model. The t-value represents a test of each independent variable individually. Beta refers to the standardized regression coefficient and is an indicator that can be used to compare the magnitude of the impact relationship. The results indicated that the significant regression equation, with F = 75.231, R^2^ = 0.321, β = 0.567, t = 8.674, *P* < 0.01, which demonstrated that physical exercise with additional visual tasks could account for 32.1% of the variation in KVA. Furthermore, physical exercise with additional visual tasks had a significant positive effect on KVA.

A linear regression analysis was conducted with physical exercise with additional visual tasks as the independent variable and left eye UDVA (difference scores) as the dependent variable. The results indicated that a significant regression equation, F = 35.384, Beta = 0.427, t = 5.948, *P* < 0.01, suggesting that physical exercise with additional visual tasks has a significant positive impact on left eye uncorrected distance vision. The results indicated that the significant regression equation, with F = 75.231, R^2^ = 0.182, β = 0.567, t = 8.674, *P* < 0.01, which demonstrated that physical exercise with additional visual tasks could account for 18.2% of the variation in left eye UDVA. Furthermore, physical exercise with additional visual tasks had a significant positive effect on left eye UDVA.

A linear regression analysis was conducted with physical exercise with additional visual tasks and KVA (difference scores) as independent variables, and left eye UDVA (difference scores) as the dependent variable. The results indicated that the structural equation model was valid, with R^2^ = 0.211, F = 22.394, *P* < 0.01, which demonstrated that physical exercise with additional visual tasks and KVA could explain 21.1% of the variation in left eye UDVA. Furthermore, it indicated that at least one of the factors—physical exercise with additional visual tasks or KVA—exerted an effect on left eye UDVA. The analysis of physical exercise with additional visual tasks and left eye UDVA showed that the standardized regression coefficient for physical exercise with additional visual tasks was Beta = 0.291, t = 3.415, *P* = 0.001, *P* < 0.01, indicating a significant regression equation. This suggested that physical exercise with additional visual tasks has a significant positive impact on left eye UDVA. Additionally, the regression analysis of KVA and left eye UDVA revealed that the standardized regression coefficient for KVA was Beta = 0.239, t = 2.806, *P* = 0.006, *P* < 0.05. This also indicated a significant regression equation, demonstrating that KVA has a significant positive impact on left eye UDVA.

A linear regression analysis was conducted with physical exercise with additional visual tasks, KVA (difference scores), and left eye UDVA (difference scores) as independent variables, and anxiety (difference scores) as the dependent variable. The results indicated that the structural equation model was valid, with R^2^ = 0.290, F = 21.359, and *P* < 0.01, which demonstrated that physical exercise with additional visual tasks, left eye UDVA, and KVA could explain 29.0% of the variation in anxiety. Furthermore, it indicated that at least one of these factors—physical exercise with additional visual tasks, left eye UDVA, or KVA—could exert an effect on anxiety. Furthermore, testing for multicollinearity within the model revealed that all VIF values were below 5, indicating no multicollinearity issues. Meanwhile, the D-W values clustered around 2, suggesting no autocorrelation exists within the model. This confirms that no correlation exists between the sample data, indicating a robust model. The analysis of physical exercise with additional visual tasks and anxiety showed that the standardized regression coefficient for physical exercise with additional visual tasks was Beta = −0.240, t = −2.833, *P* = 0.005, *P* < 0.01, indicating a significant regression equation. This suggested that physical exercise with additional visual tasks has a significant negative impact on anxiety. Furthermore, the regression analysis of KVA and anxiety revealed that the standardized regression coefficient for KVA was Beta = −0.219, t = −2.614, *P* = 0.010, *P* < 0.05. This indicated a significant regression equation, demonstrating that KVA has a significant negative impact on anxiety. Lastly, the regression analysis of left eye UDVA and anxiety showed that the standardized regression coefficient for left eye UDVA was Beta = −0.211, t = −2.774, *P* = 0.006, *P* < 0.01, indicating a significant regression equation. This suggested that left eye UDVA has a significant negative impact on anxiety.

#### 3.3.2 Chain mediation effect of kinetic visual acuity and left eye uncorrected distance visual acuity

The results of the chain mediation effect analysis of kinetic visual acuity (KVA) and left eye uncorrected distance visual acuity (UDVA) are presented in Table 8. The direct effect of physical exercise incorporating additional visual tasks on anxiety was significant, with an effect size of −0.240 and a 95% confidence interval that did not include zero. This direct effect accounted for 52.9% of the total effect. The total indirect effect, which includes all indirect pathways, was −0.454, with a 95% confidence interval that did not include zero, indicating a significant total indirect effect. Indirect Effect 1 (Physical Exercise with Additional Visual Tasks→*KVA*→Anxiety) was significant, with an effect size of −0.124 and a 95% confidence interval that did not include zero, accounting for 27.3% of the total effect. Indirect Effect 2 (Physical Exercise with Additional Visual Tasks→Left Eye UDVA→Anxiety) was also significant, with an effect size of −0.062 and a 95% confidence interval that did not include zero, accounting for 13.7% of the total effect. Indirect Effect 3 (Physical Exercise with Additional Visual Tasks→*KVA*→Left Eye UDVA→Anxiety) was significant as well, with an effect size of −0.029 and a 95% confidence interval that did not include zero, accounting for 6.4% of the total effect. These results indicated that both KVA and left eye UDVA serve as significant chain mediators in the relationship between physical exercise with additional visual tasks and anxiety.

**TABLE 8:** Chain mediation effect of kinetic visual acuity and left eye uncorrected distance visual acuity.

Type of effect	Effect size	Boot SE	Lower limit	Upper limit	Proportion of effect size
Direct effect	–0.240	0.085	–0.405	–0.074	52.9%
Indirect effect 1	–0.124	0.043	–0.212	–0.043	27.3%
Indirect effect 2	–0.062	0.032	–0.135	–0.012	13.7%
Indirect effect 3	–0.029	0.015	–0.061	–0.005	6.4%
Total indirect effect	–0.454	0.071	–0.592	–0.315	–

**FIGURE 6:**
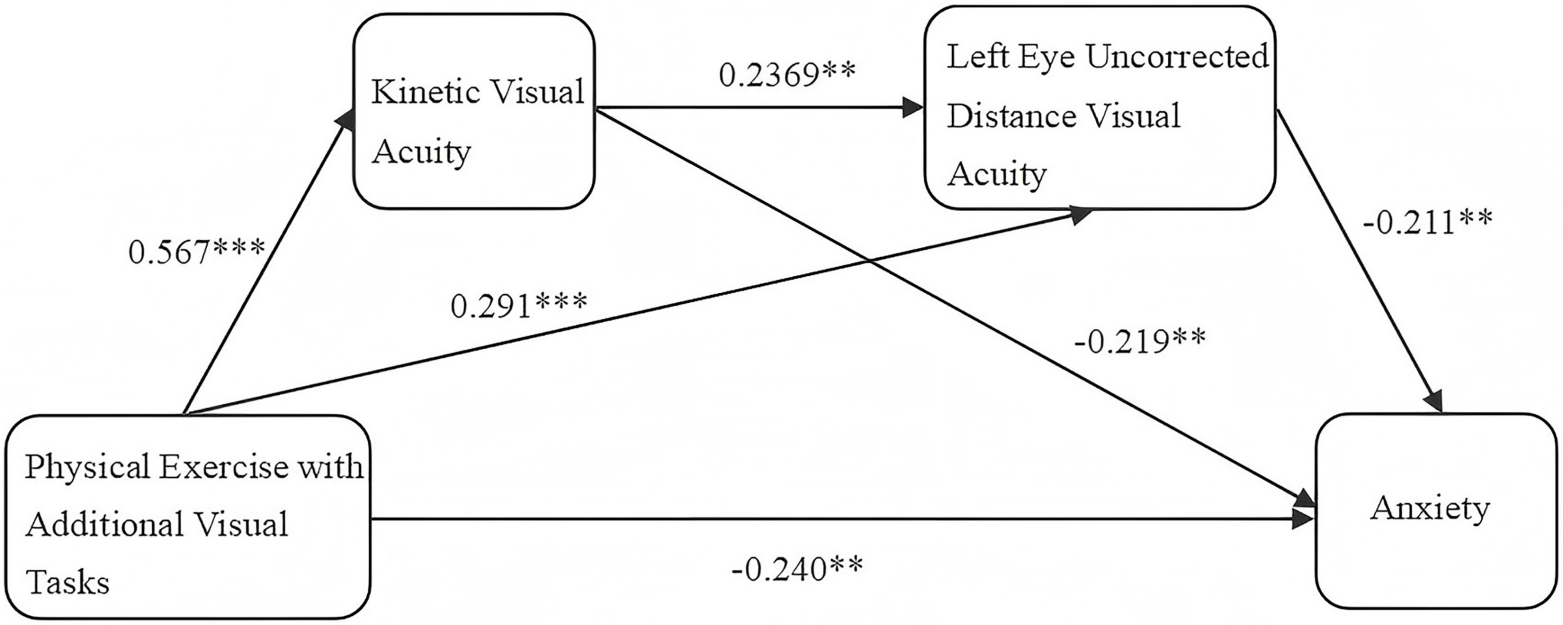
Chain mediation model of kinetic visual acuity and left eye uncorrected distance visual acuity. **Indicates *P* < 0.01, representing a significant difference; ***Indicates *P* < 0.001, representing a highly significant difference.

The chained mediation effect model of kinetic visual acuity (KVA) and left eye uncorrected distance visual acuity (UDVA) is shown in Figure 6. The Figure 6 illustrated that physical exercise incorporating additional visual tasks has a direct effect on anxiety disorders among fifth-grade students, indicating that such exercise can significantly alleviate students’ anxiety disorders. KVA partially mediated the effect of this exercise on students’ anxiety, suggesting that physical exercise with additional visual tasks can reduce anxiety disorders by improving students’ KVA. Similarly, left eye UDVA also partially mediated the effect, implying that the exercise can mitigate anxiety disorders by enhancing students’ left eye UDVA. Regression analysis showed that the regression coefficient of KVA on left eye UDVA is 0.239, indicating a significant positive relationship between KVA and left eye UDVA.

The Figure 2 indicated that the path effect from physical exercise incorporating additional visual tasks through KVA and left eye UDVA to anxiety is significant, establishing the chained mediation effect. This implied that participation in physical exercise with additional visual tasks can significantly enhance students’ KVA, which in turn improves their left eye UDVA, thereby alleviating their anxiety disorders. These results suggested that KVA and left eye UDVA serve as chained mediators in the impact of physical exercise incorporating additional visual tasks on anxiety disorders among fifth-grade students.

#### 3.3.3 Regression analysis of physical exercise with additional visual tasks, KVA, right eye UDVA, and total anxiety score

A linear regression analysis was conducted with physical exercise with additional visual tasks as the independent variable and right eye UDVA (difference scores) as the dependent variable. The results indicated that the significant regression equation, with F = 75.231, R^2^ = 0.231, F = 47.691, Beta = 0.480, t = 6.906, *P* < 0.01, which demonstrated that physical exercise with additional visual tasks could account for 23.1% of the variation in right eye UDVA. Furthermore, physical exercise with additional visual tasks had a significant positive effect on right eye UDVA.

A linear regression analysis was conducted with physical exercise with additional visual tasks and KVA (difference scores) as independent variables, and right eye UDVA (difference scores) as the dependent variable. The results indicated that the structural equation model was valid, with R^2^ = 0.255, F = 26.995, *P* < 0.01, which demonstrated that physical exercise with additional visual tasks and KVA could explain 25.5% of the variation in right eye UDVA. Furthermore, it indicated that at least one of the factors—physical exercise with additional visual tasks or KVA—exerted an effect on right eye UDVA. The analysis of physical exercise with additional visual tasks and right eye UDVA showed that the standardized regression coefficient for physical exercise with additional visual tasks was Beta = 0.347, t = 4.448, *P* = 0.000, *P* < 0.01, indicating a significant regression equation. This suggested that physical exercise with additional visual tasks has a significant positive impact on right eye UDVA. Additionally, the regression analysis of KVA and right eye UDVA revealed that the standardized regression coefficient for KVA was Beta = 0.188, t = 2.253, *P* = 0.026, *P* < 0.05. This also indicated a significant regression equation, demonstrating that KVA has a significant positive impact on right eye UDVA.

A linear regression analysis was conducted with physical exercise with additional visual tasks, KVA (difference scores), and right eye UDVA (difference scores) as independent variables, and anxiety (difference scores) as the dependent variable. The results indicated that the structural equation model was valid, with R^2^ = 0.266, F = 19.009, and *P* < 0.01, which demonstrated that physical exercise with additional visual tasks, right eye UDVA, and KVA could explain 26.6% of the variation in anxiety. Furthermore, it indicated that at least one of these factors—physical exercise with additional visual tasks, right eye UDVA, or KVA—could exert an effect on anxiety. Furthermore, testing for multicollinearity within the model revealed that all VIF values were below 5, indicating no multicollinearity issues. Meanwhile, the D-W values clustered around 2, suggesting no autocorrelation exists within the model. This confirms that no correlation exists between the sample data, indicating a robust model. The analysis of physical exercise with additional visual tasks and anxiety showed that the standardized regression coefficient for physical exercise with additional visual tasks was Beta = −0.255, t = −2.894, *P* = 0.004, *P* < 0.01, indicating a significant regression equation. This suggested that physical exercise with additional visual tasks has a significant negative impact on anxiety. Furthermore, the regression analysis of KVA and anxiety revealed that the standardized regression coefficient for KVA was Beta = −0.246, t = −2.918, *P* = 0.004, *P* < 0.05. This indicated a significant regression equation, demonstrating that KVA has a significant negative impact on anxiety. Lastly, the regression analysis of right eye UDVA and anxiety showed that the standardized regression coefficient for right eye UDVA was Beta = −0.124, t = −1.562, *P* = 0.120, *P* > 0.05, indicating that there was no significant difference in the regression equation. This suggested that right eye UDVA did not have an impact on anxiety.

#### 3.3.4 Chain mediation effect of kinetic visual acuity and right eye uncorrected distance visual acuity

The chain mediation effect of kinetic visual acuity (KVA) and right eye uncorrected distance visual acuity (UDVA) is presented in Table 9. The direct effect of physical exercise incorporating additional visual tasks on anxiety was significant, with an effect value of −0.255 and a 95% confidence interval excluding 0, accounting for 56.2% of the effect size. The total indirect effect value was −0.454, with a 95% confidence interval excluding 0, indicating a significant total indirect effect. The first indirect effect (physical exercise with additional visual tasks→*KVA*→anxiety) was significant, with an effect value of −0.139, and a 95% confidence interval excluding 0, accounting for 30.6% of the effect size. The second indirect effect (physical exercise with additional visual tasks→right eye UDVA→anxiety) was not significant, with an effect value of −0.046, and a 95% confidence interval including 0, accounting for 10.1% of the effect size. The third indirect effect (physical exercise with additional visual tasks→*KVA*→right eye UDVA→anxiety) was not significant, with an effect value of −0.013, and a 95% confidence interval including 0, accounting for 2.9% of the effect size. In summary, the chain mediation effect of KVA and right eye UDVA on the impact of physical exercise incorporating additional visual tasks on anxiety was not significant.

**TABLE 9:** Chain mediation effect analysis of kinetic visual acuity and right eye uncorrected distance visual acuity.

Mediation pathway	Effect size	Boot SE	Lower limit	Upper limit	Proportion of effect size
Direct effect	–0.255	0.088	–0.428	–0.082	56.2%
Indirect effect 1	–0.139	0.043	–0.225	–0.058	30.6%
Indirect effect 2	–0.046	0.034	–0.117	0.016	10.1%
Indirect effect 3	–0.013	0.011	–0.039	0.005	2.9%
Total indirect effect	–0.454	0.071	–0.592	–0.315	–

## 4 Discussion

### 4.1 Analysis of the current status of vision and anxiety in 10–11-year-old children

The results of this study indicated that, prior to the experimental intervention, the average UDVA for both left and right eyes in the experimental group was 4.80, with an average KVA of 0.36. In the control group, the average UDVA for the left and right eyes were 4.79 and 4.78, respectively, with an average KVA of 0.37, consistent with the findings of Yin et al. (2022a). When a child over 6 years old has uncorrected distance visual acuity below 5.0, it indicates poor uncorrected distance vision. This demonstrated that the visual acuity status among fifth-grade students was concerning, highlighting the urgency of myopia prevention and control.

This study involved 161 participants. Prior to the experimental intervention, screening for anxiety was conducted using the Childhood Anxiety Sensitivity Index, revealing that 114 participants (approximately 70.81%) exhibited signs of anxiety. This suggested a high prevalence of anxiety disorders among the study participants, consistent with the findings of Gu et al. (2013), Li et al. (2022). Chinese city norms suggested that the SCARED total score > 23 indicates a potential anxiety disorder, which should be further evaluated in conjunction with clinical diagnosis. Research findings indicated that fifth-grade students exhibited a disproportionately high prevalence of anxiety disorders and a widespread increase in anxiety levels. This may stem from intensifying social competition in contemporary China, where increasing pressures from society, schools, and families are exerted upon children and adolescents. Concurrently, upper elementary students, as they age, enter a phase of rapid self-awareness development. They face not only academic pressures associated with the transition to middle school but also peer evaluations, comparisons among classmates, and parental expectations. These factors can easily lead to feelings of confusion and anxiety, as well as heightened sensitivity to criticism and setbacks. At the same time, this may also be related to the students’ mental state at the time, such as whether they were facing exams or important performances in the near future.

It can be seen that the situation regarding the visual health of children and adolescents in China today, which is primarily characterized by myopia, and their mental health, which is primarily characterized by anxiety, is becoming increasingly severe. Society, schools, and families must pay close attention and work together to jointly safeguard the visual and mental health of children and adolescents.

### 4.2 The impact of physical exercise with additional visual tasks on vision and anxiety in 10–11-year-old children

The study results indicated that both the control and experimental groups showed a significant improvement in UDVA in both eyes after the experimental intervention. Notably, the improvement in UDVA was significantly greater in the experimental group than in the control group. This suggested that physical exercise can enhance UDVA in fifth-grade students, and incorporating additional visual tasks into the exercise regimen yields even better results. Moreover, there was a significant improvement in KVA in the experimental group compared to pre-intervention levels, and this improvement was significantly greater than that observed in the control group. These findings indicated that physical exercise with additional visual tasks positively affects enhancing students’ KVA.

Research has shown that combining physical exercise with ciliary muscle training for near-far accommodation can effectively reduce the stress of the eyes remaining in a fixed position for extended periods, thereby alleviating eye fatigue. Additionally, this approach helps exercise the eye muscles, enhancing their adaptability and resilience, and reducing the occurrence of eye fatigue (Adam, 2016). The sclera, the outer layer of the eye, encases and protects its internal structures. Scleral collagen, a crucial component of the sclera, provides support and protection for the eye. The ciliary muscle controls the shape of the lens and adjusts the eye’s focus at different distances. Physical activity has shown positive effects in promoting blood circulation, accelerating scleral collagen synthesis, and enhancing the sclera’s support for the ciliary muscle’s lens adjustments (Metlapally and Wildsoet, 2015). By increasing the ciliary muscle’s accommodative response and reducing accommodative lag, this approach can effectively slow the progression of myopia (Yue et al., 2013). Furthermore, research in cognitive neuroscience has shown that engaging in physical exercise may release endorphins (Dishman and O’Connor, 2009) and stimulate the growth of brain capillaries (Kleim et al., 2002), thereby contributing to improved mental health. These may represent the physiological mechanisms through which physical exercise with additional visual tasks can positively effect children’s vision and anxiety levels.

After a 16 weeks intervention, the total scores on the anxiety scale for both the control and experimental groups showed significant differences compared to pre-intervention scores. Furthermore, post-intervention scores for the experimental group were significantly lower than those for the control group. This indicated that physical exercise can effectively alleviate anxiety disorders in 10–11 years-old children, with physical exercise incorporating additional visual tasks yielding even better results. Previous research has found that participation in physical exercise can enhance physical fitness and cardiorespiratory function, thereby reducing the risk of psychological disorders (Kandola et al., 2019). Additionally, studies had shown that physical exercise can effectively treat depression as part of clinical therapies for mental health issues (Schuch et al., 2018).

### 4.3 The relationship between kinetic visual acuity, uncorrected distance visual acuity, and anxiety in 10–11-year-old children

This study found significant positive correlations between UDVA of both eyes and KVA. Additionally, significant negative correlations were observed between the total scores on the anxiety scale and both UDVA and KVA for both eyes. Previous research indicated a moderate positive correlation between kinetic and static visual acuity (Sun et al., 2018). Zhou et al. (2020) suggested that KVA can predict UDVA. A review reports a stronger association between myopia and anxiety than between myopia and depression, noting that the severity of refractive errors increases with higher levels of anxiety and depression (You et al., 2022). Another study found a negative correlation between refractive error and emotional dimension scores, indicating that higher levels of refractive error are associated with corresponding emotional dimension scores (Wu et al., 2022).

However, owing to the cross-sectional design of the study, the observed associations could not infer causality and may be constrained by bidirectionality and unmeasured confounding factors. Regarding the relationship between anxiety and vision, it may be possible for anxiety to cause a decline in visual acuity. However, an equally plausible explanation may be that children with poorer vision are more prone to developing anxiety. Both scenarios demonstrated a close association between anxiety and vision. Moreover, the association between anxiety and visual acuity may be influenced by potential confounding variables. For instance, students’ screen time could serve as both a risk factor for declining vision and a correlate of heightened anxiety symptoms. However, this study primarily examined the direct link between visual acuity and anxiety without collecting data on other indicators, which may have impacted the findings. Despite these limitations, the results still have value in identifying an association between anxiety and visual acuity.

The observed negative correlations between kinetic visual acuity (KVA), uncorrected distance visual acuity (UDVA), and anxiety in children may be attributed to the general trends in psychological health and refractive development. Previous research indicated that myopia is preventable and manageable, it is not reversible and can lead to further visual deterioration and increased psychological stress. Initially, children experience minimal psychological stress due to declining vision, but sustained stress can disrupt the autonomic nervous system (ANS). Since the ANS regulates the eye function, heightened psychological stress can cause ciliary muscle spasms, reducing the muscle’s sensitivity and function, leading to blurred retinal images and accelerated myopia progression. Additionally, rapid vision decline creates more psychological stress, forming a vicious cycle (Sabel et al., 2018). Without timely intervention, this can result in complications such as glaucoma and cataracts. The rapid vision decline negatively impacts students’ lives and learning, causing worry, anxiety, and even fear. Prolonged psychological stress may lead to depression and other mental health issues (Li, 2020). Beyond myopia, other visual problems such as amblyopia and strabismus can adversely affect adolescents’ psychological health. Children with poor vision may face ridicule and isolation from peers, leading to social withdrawal, reluctance to participate in outdoor activities, increased introversion, and heightened sensitivity to external feedback. Breaking the vicious cycle between poor vision and psychological problems is crucial to improving both visual and psychological health in children and adolescents.

### 4.4 The chain mediation effect of kinetic visual acuity and left eye uncorrected distance visual acuity in the impact of physical exercise with additional visual tasks on student anxiety

The results of this study, obtained through the Bootstrap method, indicated that the impact path of physical exercise with additional visual tasks—KVA—left eye UDVA—anxiety is significant. This confirmed the chain mediation effect of KVA and left eye UDVA in the influence of physical exercise with additional visual tasks on anxiety. Regression analysis showed that KVA can significantly and positively predict students’ left eye UDVA, indicating that improvement in KVA was associated with enhancement in left eye UDVA. Experimental findings by Cao et al. (2019) demonstrate a trend of increasing kinetic visual sensitivity from pre-test to mid-test, suggesting that kinetic visual sensitivity changes prior to static visual sensitivity. Cai’s (2022) research indicateed a high degree of correlation between KVA and UDVA, with KVA having a certain predictive validity for UDVA.

This indicated that physical exercise incorporating additional visual tasks can significantly alleviate student anxiety directly and also through the mediating effects of KVA and left eye uncorrected UDVA. Among these, KVA showed a significant improvement first, followed by a notable enhancement in left eye UDVA, leading to a reduction in students’ anxiety levels.

The chain mediating effect of KVA and left eye UDVA in the impact of physical exercise with additional visual tasks on student anxiety further confirmed that UDVA improves with the enhancement of KVA. This suggested that when designing interventions for myopia prevention and control in children and adolescents, it is crucial to emphasize KVA training and monitor changes in KVA indicators to better predict the development and changes in UDVA. Additionally, the close relationship between KVA, UDVA, and anxiety should be considered. Schools, physical education teachers, and parents need to promptly monitor changes in children’s vision and mental health indicators during their growth and development, ensuring early detection, prevention, intervention, and rehabilitation. Furthermore, the value of physical exercise, particularly exercises incorporating ciliary muscle adjustment training, should be emphasized for its positive effects on both vision and mental health.

### 4.5 The cause for the non-establishment of the chain mediation effect of kinetic visual acuity and right eye uncorrected distance visual acuity in the impact of physical exercise with additional visual tasks on student anxiety

The human eye is divided into a dominant eye and a non-dominant eye, which differ in their visual functions. However, whether the dominant eye is the left or right eye is not fixed for every individual. The dominant eye, also known as the primary eye, leading eye, or main eye, plays a leading role in binocular fixation activities. It receives more visual information input and is responsible for localization and analysis. The non-dominant eye is also referred to as the secondary eye or auxiliary eye. The visual information acquisition capabilities of the two eyes exhibit an imbalance. The visual quality and optic nerve response of the non-dominant eye are inferior to those of the dominant eye. During binocular visual activities, the dominant eye handles the task of identifying the distance of objects and produces clearer images, while the non-dominant eye operates in a subordinate role, responsible for recognizing the background surrounding the target object. Research findings indicated that the chain mediated effect of KVA and right eye UDVA in the impact of physical exercise with additional visual tasks on student anxiety was not established. This may be related to the dominant eye of the participating students and the sample size of the experiment.

## 5 Conclusion

Physical exercise with additional visual tasks can effectively improve both UDVA and KVA in 10–11 years-old children, while also alleviating anxiety. Anxiety levels in these children showed significant negative correlations with both UDVA and KVA before and after the intervention. KVA had significant positive correlations with left eye UDVA before and after the experiment, whereas it was not significantly correlated with right eye UDVA before the experiment but became significantly positively correlated after. KVA and left eye UDVA serve as chain mediators in the impact of physical exercise incorporating additional visual tasks on the anxiety levels of 10–11 years-old children.

## 6 Limitations and future directions

The sample size in this study was limited. Due to the study’s long-term and complex nature, only a subset of fifth-grade students was included. Future studies should aim to include larger and more diverse samples to increase generalizability.

Cluster randomization may generate clustering effects. Students within the same class may exhibit more similar performance than those in different classes due to shared classroom environments and social interactions. However, this study primarily focused on vision and anxiety indicators while employing physical exercise as the intervention. Such similarities may not be pronounced in this context but could limit the generalizability of findings to broader student populations. Future research should attempt to validate the universality of these findings across more diverse schools and classes.

Students were assessed using the SCARED self-report scale. Although a series of supervision measures were implemented during testing, self-report bias may still exist. Students may underreport anxiety levels due to social desirability effects or exhibit variations in understanding the scale items. While self-report scales are commonly used tools in childhood anxiety research, future studies could achieve more comprehensive and objective intervention effect evaluations by employing triangulation with multi-source information such as parent reports, teacher assessments, or clinical interviews.

This study focused exclusively on uncorrected distance visual acuity and kinetic visual acuity as measures of visual function, neglecting other important ocular parameters. Future research should explore additional factors such as axial length, anterior chamber depth, corneal curvature, refractive error, and intraocular pressure to provide a more comprehensive assessment of visual health.

The intervention period of the experiment lasted 16 weeks, with a relatively short follow-up duration. The current findings only demonstrated that physical exercise with additional visual tasks yields positive short-term benefits, but the long-term sustainability of these effects remains unclear. Therefore, the conclusions of this study were limited to short-term outcomes. Future research should conduct longer-term follow-ups to assess the long-term efficacy and public health implications of this intervention.

Due to practical constraints, we were unable to rigorously control for or statistically adjust other potential lifestyle factors affecting vision and anxiety, such as daily screen time, outdoor activity duration, nutritional status, and parental involvement. These unmeasured confounders may have influenced our estimated results to some extent. For example, students in the control group may have spontaneously increased their physical exercise time, thereby diluting the differences between groups. Future research designs should strive to collect data on these covariates and adjust for them in models to more accurately estimate the net effect of the intervention.

## Data Availability

The original contributions presented in this study are included in this article/supplementary material, further inquiries can be directed to the corresponding authors.

## Footnotes

1
http://www.gpower.hhu.de/
